# Supplementary figures and images for: Biased sampling driven by bacterial population structure confounds machine learning prediction of antimicrobial resistance
Source: PLoS Biol. 2025 Dec 16;23(12):e3003539. doi: 10.1371/journal.pbio.3003539 (PMC12707637; doi:10.1371/journal.pbio.3003539)

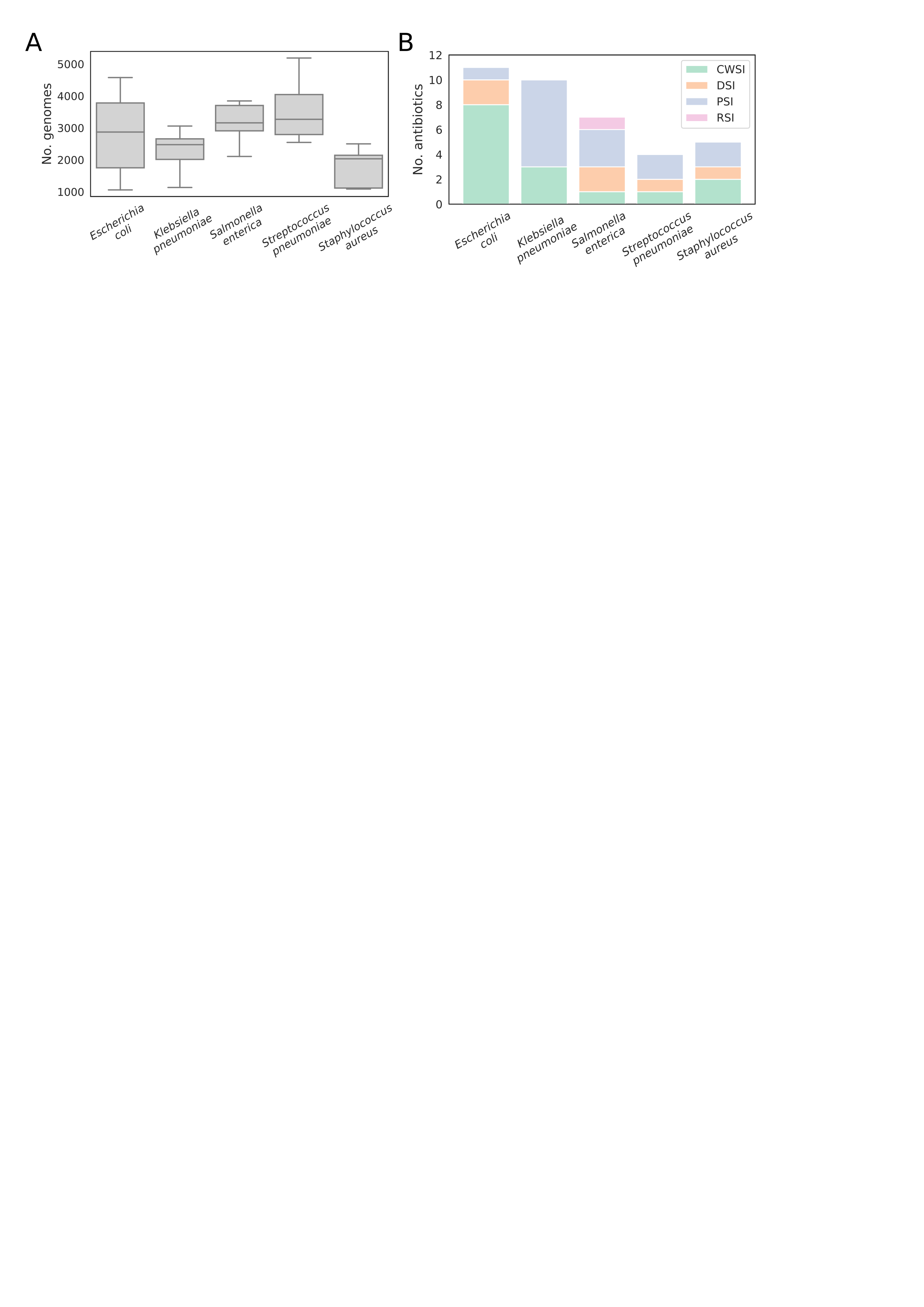

Supplement: S1 Fig — (A) The number of genomes included in the training for different antibiotics in five species. (B) The number of antibiotics in each MOA in five species. CWSI: cell wall synthesis inhibitor, DSI: DNA synthesis inhibitor, PSI: protein synthesis inhibitor, RSI: RNA synthesis inhibitor. The full list of tested antibiotics for each of the five species is included in S1 Data. (TIFF) [file pbio.3003539.s004.tiff]

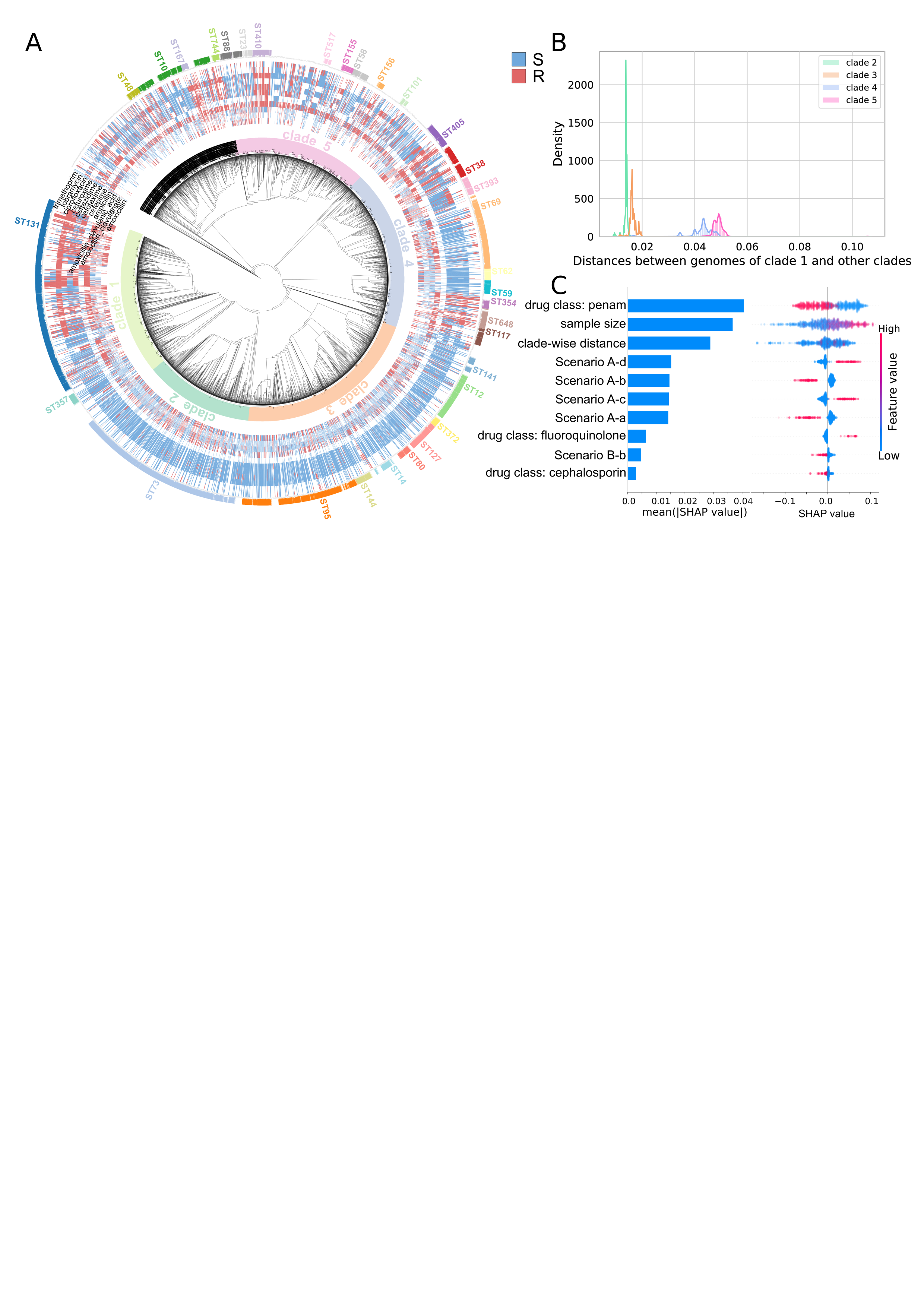

Supplement: S2 Fig — (A) Clade definition for model training, the antibiotic phenotypes, and the sequence types (ST) shown on the phylogenetic tree. S: susceptible, R: resistant. (B) The distribution of pairwise distances between genomes of clade 1 and other clades. (C) SHAP values for the top 10 features from a random forest model trained on AUC scores from both schemes A and B for E. coli. Underlying data are available in S3 Data and in the file S2_6B.tsv.gz on Mendeley Data under DOI: https://doi.org/10.17632/zs2mbjv7dn.3. (TIFF) [file pbio.3003539.s005.tiff]

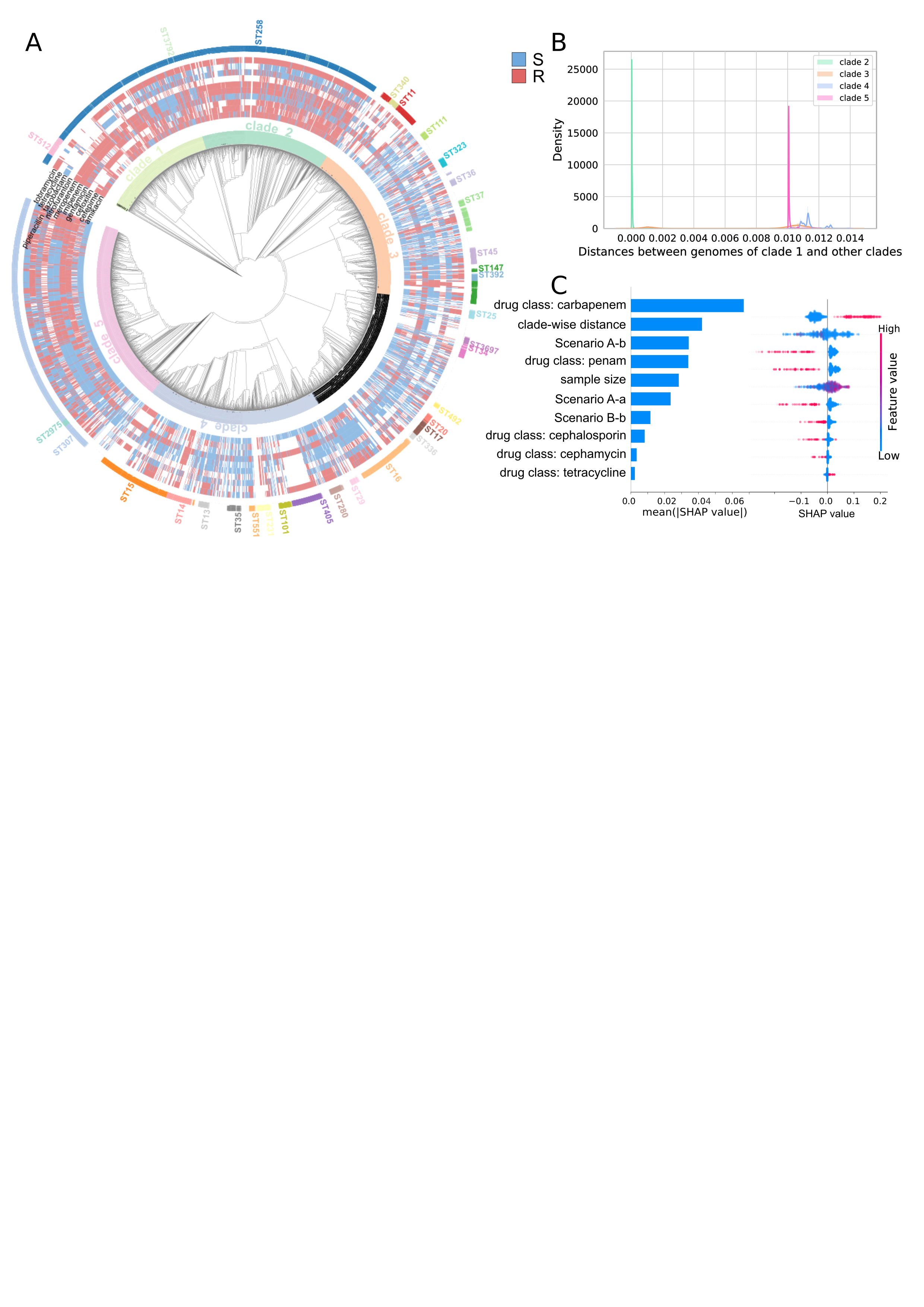

Supplement: S3 Fig — (A) Clade definition for model training, the antibiotic phenotypes, and the sequence types (ST) shown on the phylogenetic tree. S: susceptible, R: resistant. (B) The distribution of pairwise distances between genomes of clade 1 and other clades. (C) SHAP values for the top 10 features from a random forest model trained on AUC scores from both schemes A and B for K. pneumoniae. Underlying data are available in S3 Data and in the file S2_6B.tsv.gz on Mendeley Data under DOI: https://doi.org/10.17632/zs2mbjv7dn.3. (TIFF) [file pbio.3003539.s006.tiff]

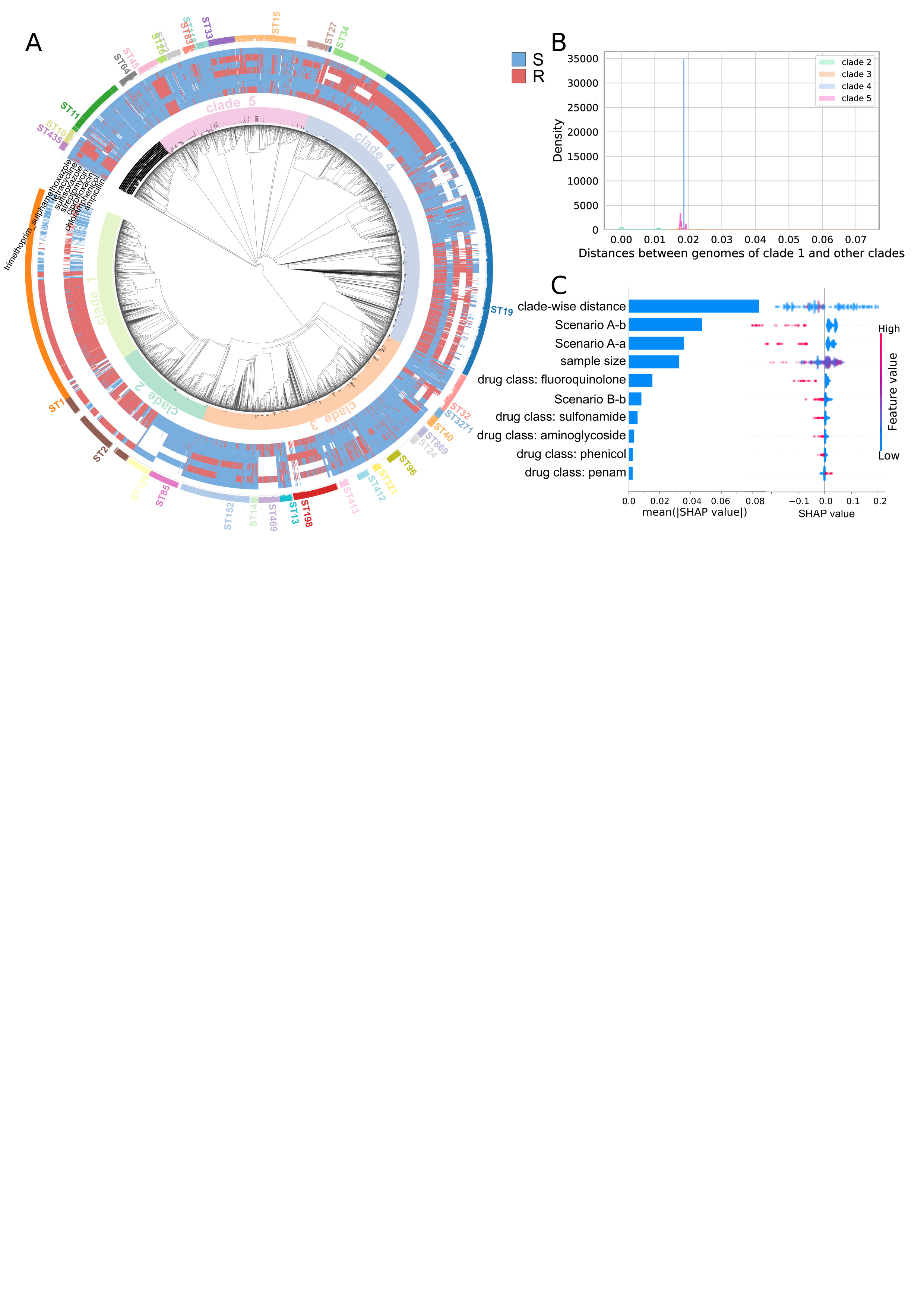

Supplement: S4 Fig — (A) Clade definition for model training, the antibiotic phenotypes, and the sequence types (ST) shown on the phylogenetic tree. S: susceptible, R: resistant. (B) The distribution of pairwise distances between genomes of clade 1 and other clades. (C) SHAP values for the top 10 features from a random forest model trained on AUC scores from both schemes A and B for S. enterica. Underlying data are available in S3 Data and in the file S2_6B.tsv.gz on Mendeley Data under DOI: https://doi.org/10.17632/zs2mbjv7dn.3. (TIFF) [file pbio.3003539.s007.tiff]

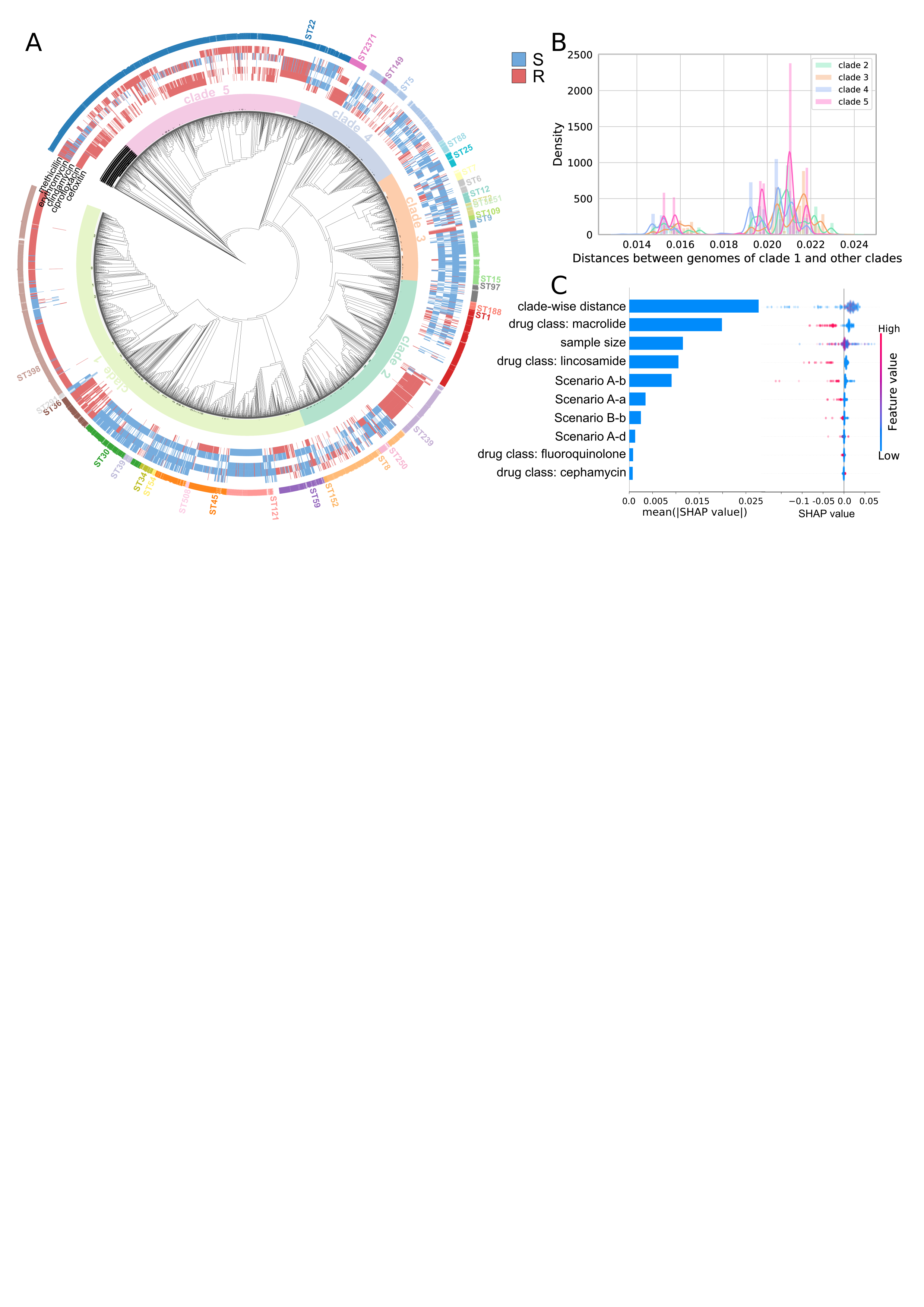

Supplement: S5 Fig — (A) Clade definition for model training, the antibiotic phenotypes, and the sequence types (ST) shown on the phylogenetic tree. S: susceptible, R: resistant. (B) The distribution of pairwise distances between genomes of clade 1 and other clades. (C) SHAP values for the top 10 features from a random forest model trained on AUC scores from both schemes A and B for S. aureus. Underlying data are available in S3 Data and in the file S2_6B.tsv.gz on Mendeley Data under DOI: https://doi.org/10.17632/zs2mbjv7dn.3. (TIFF) [file pbio.3003539.s008.tiff]

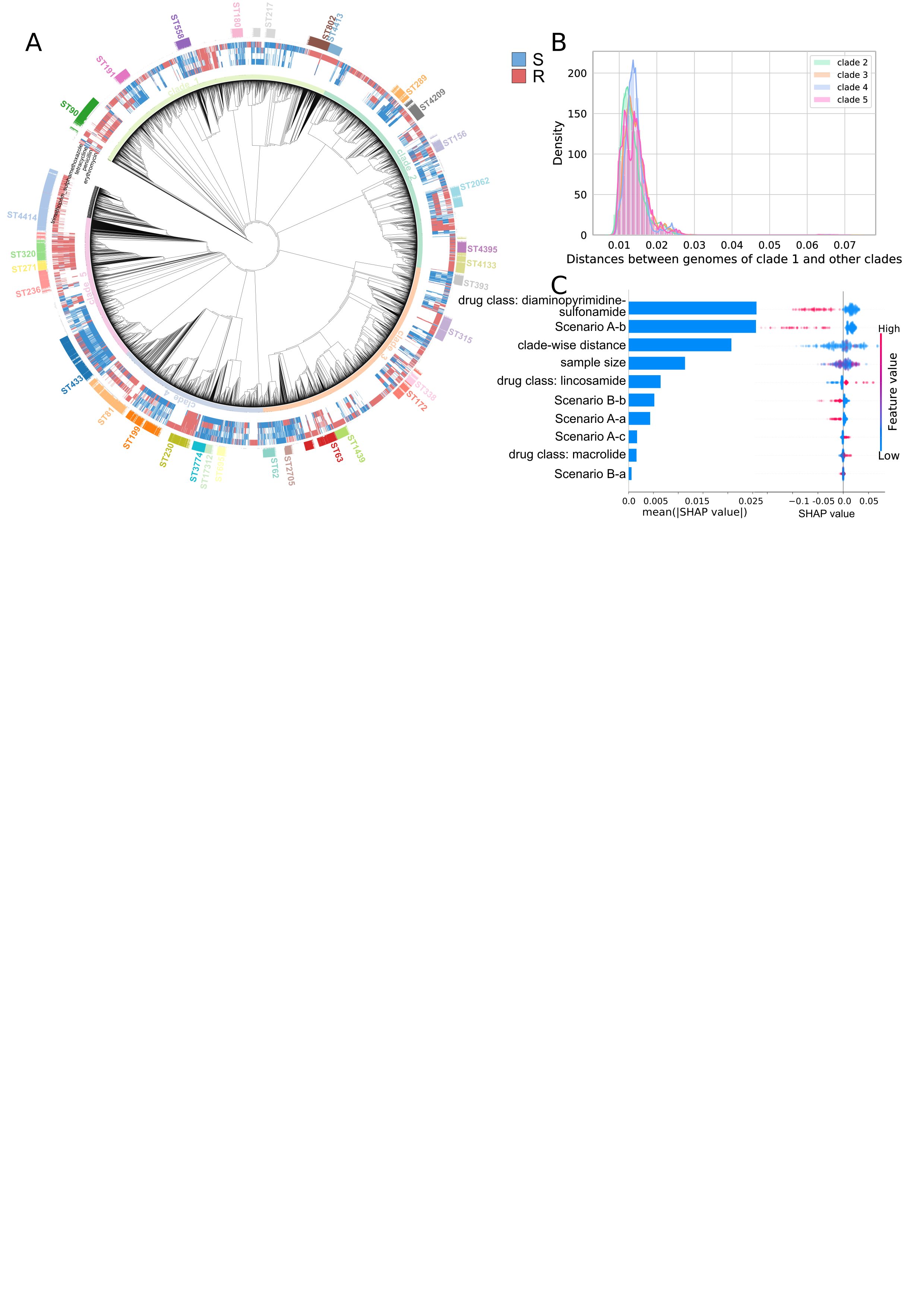

Supplement: S6 Fig — (A) Clade definition for model training, the antibiotic phenotypes, and the sequence types (ST) shown on the phylogenetic tree. S: susceptible, R: resistant. (B) The distribution of pairwise distances between genomes of clade 1 and other clades. (C) SHAP values for the top 10 features from a random forest model trained on AUC scores from both schemes A and B for S. pneumoniae. Underlying data are available in S3 Data and in the file S2_6B.tsv.gz on Mendeley Data under DOI: https://doi.org/10.17632/zs2mbjv7dn.3. (TIFF) [file pbio.3003539.s009.tiff]

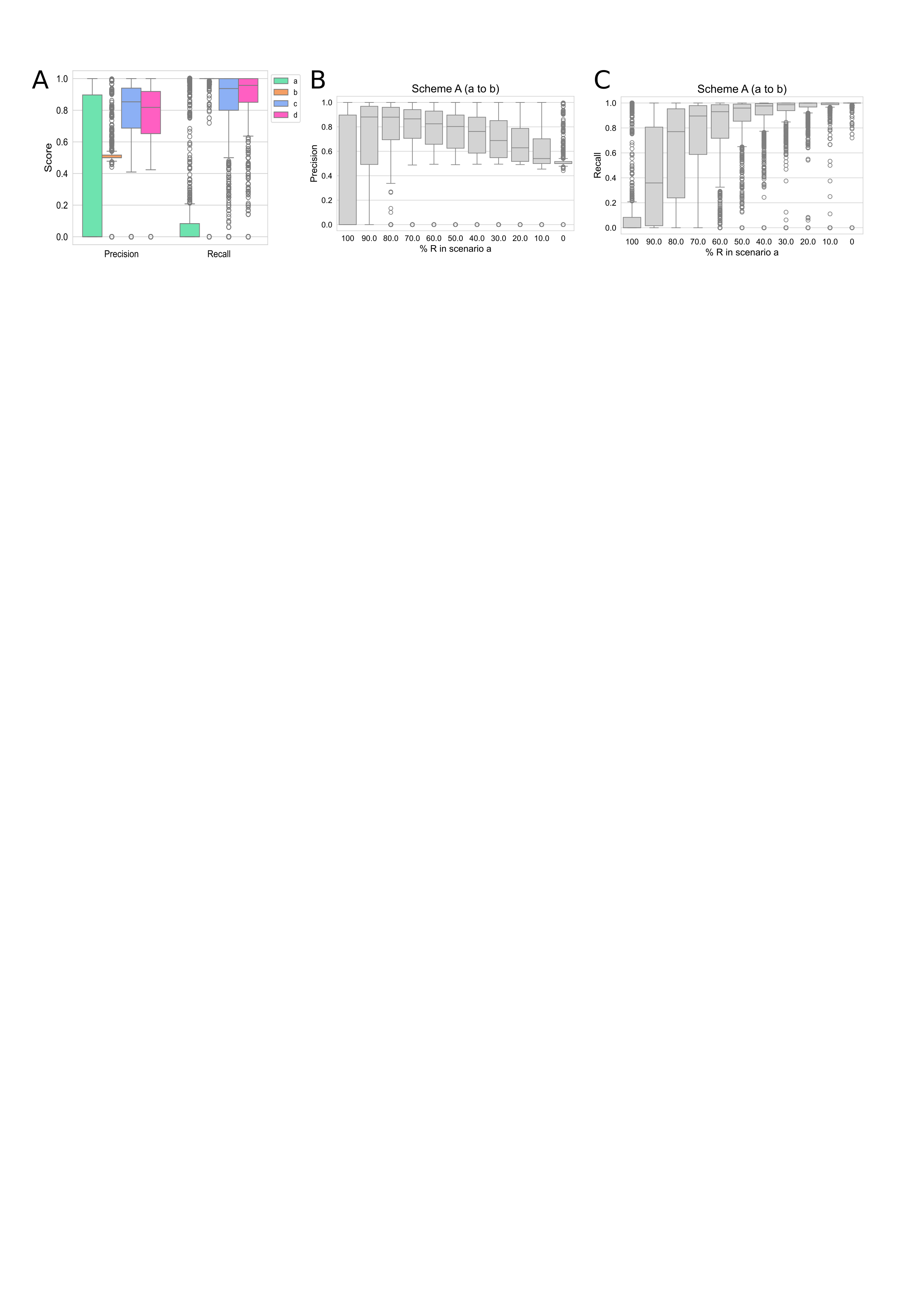

Supplement: S7 Fig — (A) Scores in each scenario for all antibiotics and species. (B) Precision scores and (C) recall scores when decreasing resistant samples and increasing susceptible samples from the paired clade were included in training, with susceptible samples considered as positives. Underlying data are available in S3 Data. (TIFF) [file pbio.3003539.s010.tiff]

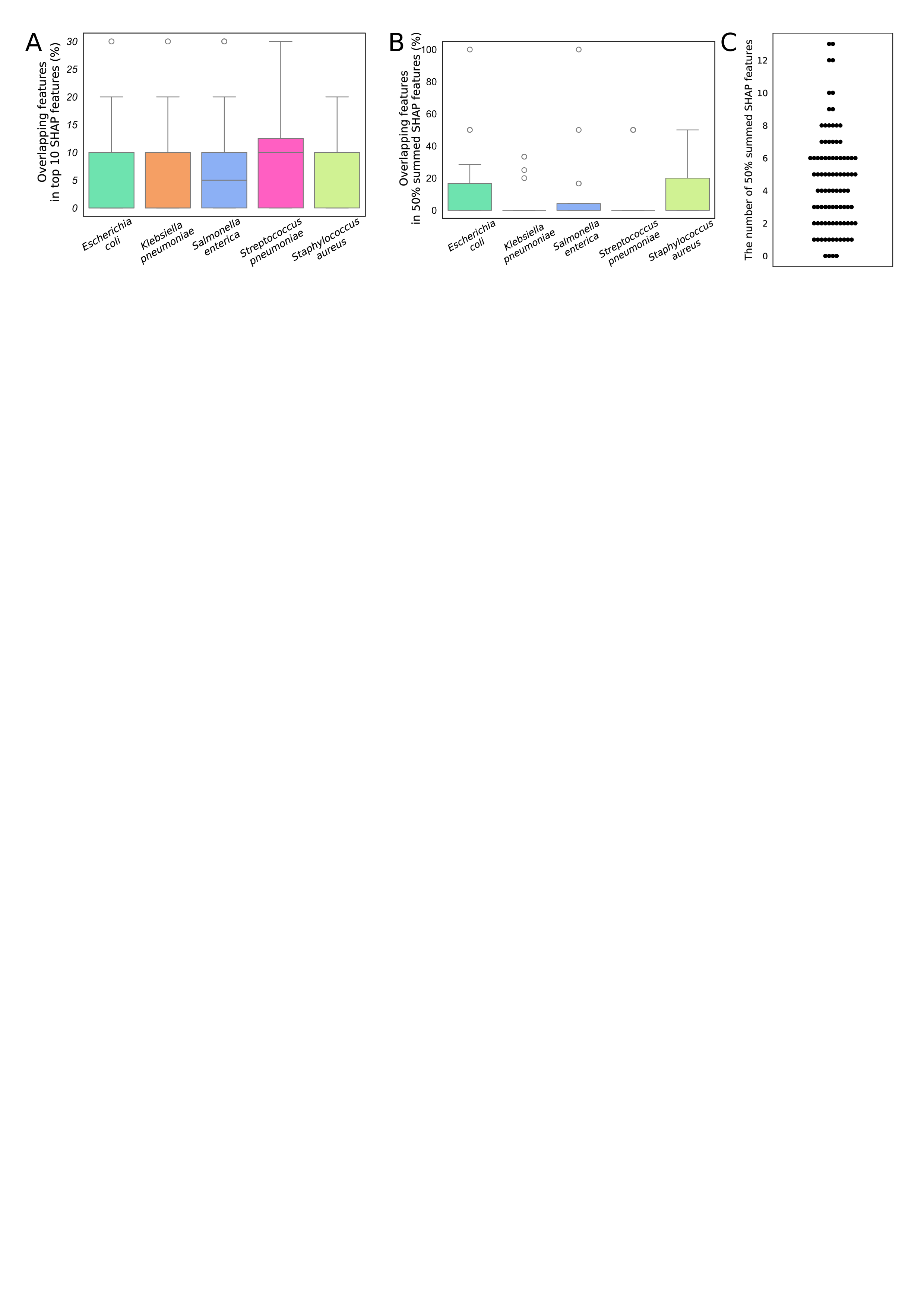

Supplement: S8 Fig — (A) top 10 features and (B) top features with SHAP values summed up to 50% of total SHAP values. (C) The distribution of the number of features with SHAP values that summed up to 50% of total SHAP values. Underlying data are available in S3 Data. (TIFF) [file pbio.3003539.s011.tiff]

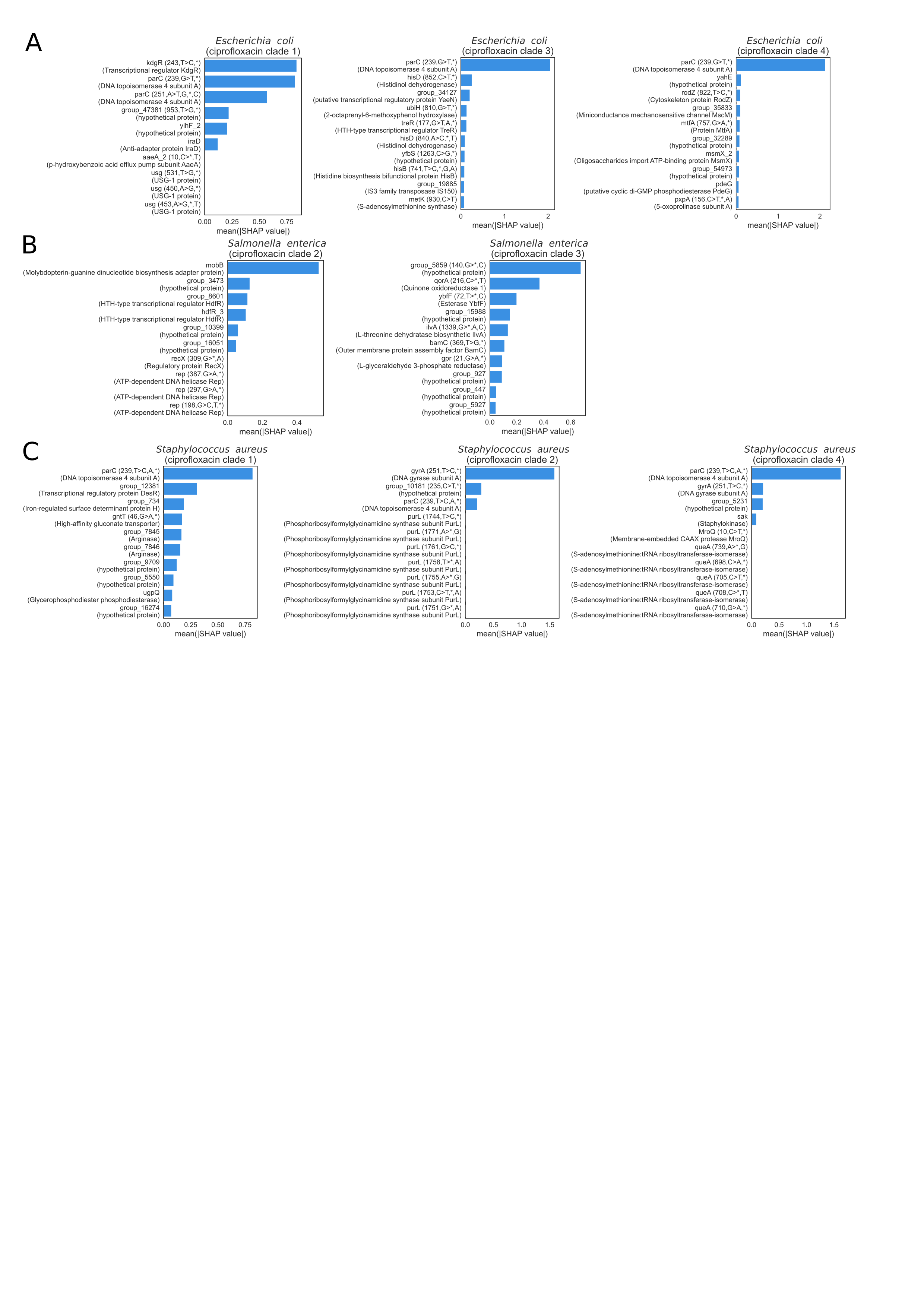

Supplement: S9 Fig — Bars indicate the average magnitude of each feature’s contribution across all strains in the clade, with higher values reflecting greater influence on model predictions. SNPs are indicated by gene names followed by the notation (position, reference allele > alternate allele(s)), while presence/absence features are indicated by gene name alone. Plots are shown for (A) Escherichia coli, (B) Salmonella enterica, and (C) Staphylococcus aureus. Only clades with at least 50 resistant and 50 susceptible strains were included. Underlying data are available in S3 Data. (TIFF) [file pbio.3003539.s012.tiff]

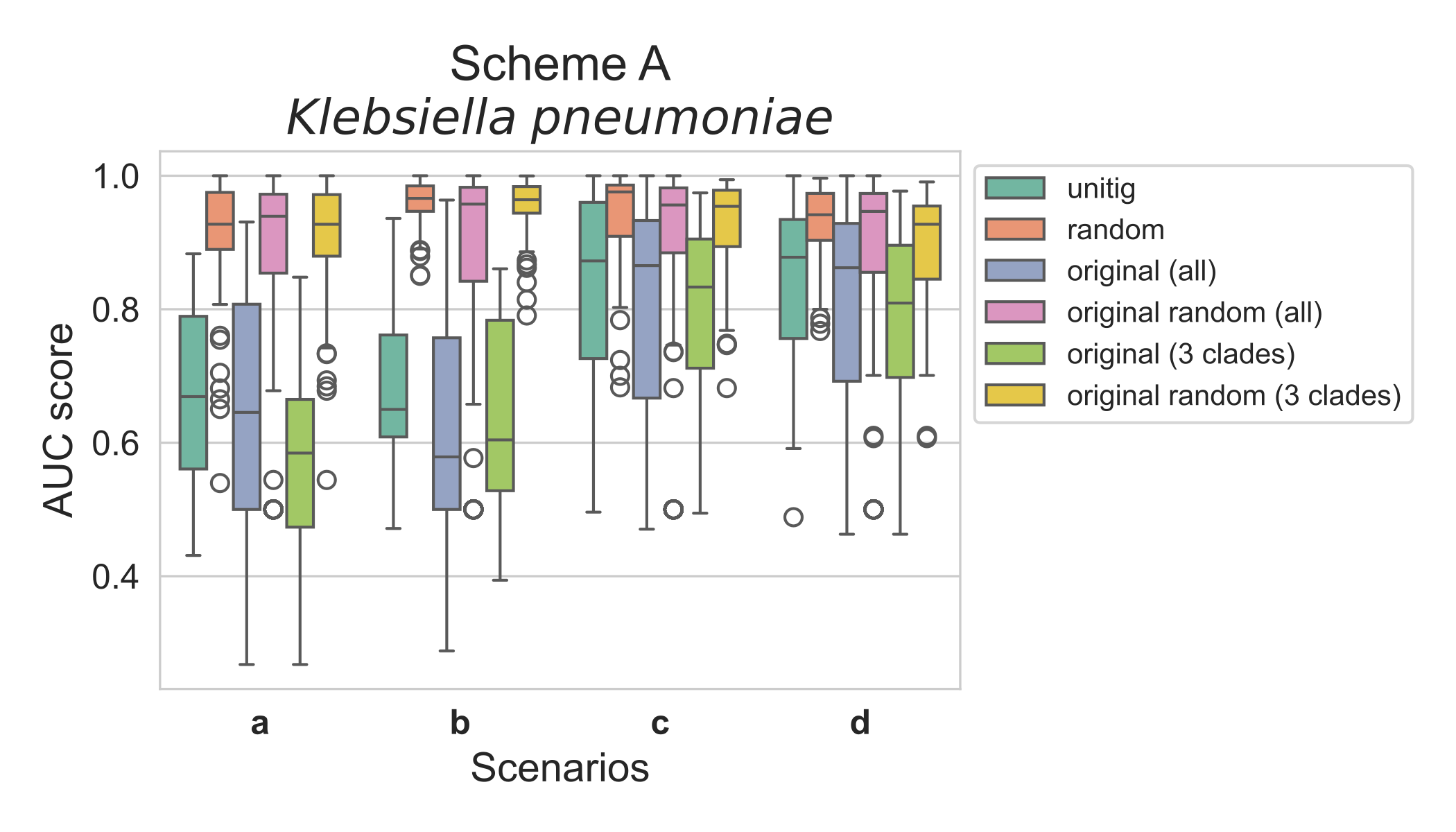

Supplement: S10 Fig — Boxplots show the distribution of the area under the ROC curve (AUC) scores for scheme A, evaluated across three clades. Models were trained using (i) presence/absence of unitigs, and (ii) the original feature set (SNPs from the core genome combined with accessory gene presence/absence). For comparison, models trained with the original SNP and accessory gene presence features were run both across all clades and across the same three clades used in the unitig analyses. Higher AUC indicates better performance. Underlying data are available in S3 Data. (TIFF) [file pbio.3003539.s013.tiff]
