# Supplementary material for: Biased sampling driven by bacterial population structure confounds machine learning prediction of antimicrobial resistance
Source: PLoS Biol. 2025 Dec 16;23(12):e3003539. doi: 10.1371/journal.pbio.3003539 (PMC12707637; doi:10.1371/journal.pbio.3003539)
Supplement: Attachment — Submitted filename: response_to_reviewers_2.pdf [file pbio.3003539.s017.pdf]

We would like to thank both the editors and reviewers for their helpful comments, and allowing us to revise our manuscript. We believe we have now addressed all outstanding comments. In response to editor comments we have modified our title to: “Biased sampling driven by bacterial population structure confounds machine learning prediction of antimicrobial resistance.” We have also revised our abstract to clarify it for a non-specialist audience. All figure data has now been collated in a new supplementary file, S3 Data, or deposited on Mendeley Data under a persistent DOI where data tables were too large to be accommodate by Excel.

Our responses to individual review comments follow.

Reviewer #1: I thank the authors for adequately tackling my comments and criticisms. I have no more remarks. The authors should check the text as there are some typos, like spaces after "-", and especially the references, several of which lack volume, page numbers and/or ms identifier. In my PDF the figures are of poor quality, bu maybe it's the site conversion to PDF that poses problem.

We thank the review for their comments, and have proof read the manuscript thoroughly. We believe the figure quality issue is due to file conversion from TIFF, as the review speculates.

Reviewer #2: The paper has not changed substantially since the previous review, with the exception of the inclusion of analyses identifying potential confounding factors and a clearer explanation of the methodological approaches used within the manuscript.

Overall, I feel that the manuscript is well positioned within the current literature to provide a valuable contribution regarding the use of machine learning without accounting for phylogenetic or genetic relatedness — particularly in the prediction of phenotypes that may be unevenly distributed across phylogenies or between bacterial species. The additional analyses provided by the authors, alongside their expanded discussion and improved contextualisation within the broader literature, have strengthened this work.

While there remain aspects of this research that could be further explored, the study as presented is sufficiently complete to be of use to the research community. This is particularly relevant given recent publications claiming improvements in antibiotic resistance prediction, some of which may suffer from the same issues and considerations highlighted by the current authors. In this context, I believe this paper is both timely and may play a useful role in the current literature base.

Minor comments:
1/ There appears to be substantial variation in the data available for sensitive/resistant (S/R) isolates across classes (i.e. many unclassified or "white" isolates in SFig 2-6). It is not clear to me why this is the case. This variation is likely a characteristic of the underlying datasets themselves, which were in some cases collected by previous authors as benchmark datasets. However, I think there is a need to address the nature fo the datasetsm, the variable sample

sizes and detail any impact this might have on the analyses performed across each split/ species/scenario and to consider how/if this variability might meaningfully influence the results.

As the reviewer suspects, this is a feature of the underlying data; the underlying data has been curated from diverse sources in the literature, and unfortunately not all strains have been tested for resistance to all antibiotics. We now detail this variability in the size of datasets on lines 69 to 72. We did not observe any clear trends in performance correlated with dataset size, so we do not believe this has had any strong impact on our results.

2/ Figures S9 and S10: The figure legends are not sufficiently detailed to explain the content of these figures.

We have revised the figure captions to clarify the content of these images.

3/ I have installed and tested the scripts in the GitHub repo on a subset of the datasets provided. I feel the repo needs slightly more annotation and updated install instructions prior to publication. I had to additionally lightgbm and ipython to the conda env described in installation instructions to ensure the scripts function effectively. Otherwise the primary script completed on a subset (E. coli, amoxicillin)

We have updated the conda environment so that it now requires lightgbm and ipython, this should resolve the issue the reviewer encountered.

4/ What is the 'shap' package/function referred to here - 'SHAP value plots were generated with the 'summary_plot' function in shap.'

We have now clarified the details of the Python SHAP package on lines 306 to 308.
