# Supplementary material for: Biased sampling driven by bacterial population structure confounds machine learning prediction of antimicrobial resistance
Source: PLoS Biol. 2025 Dec 16;23(12):e3003539. doi: 10.1371/journal.pbio.3003539 (PMC12707637; doi:10.1371/journal.pbio.3003539)
Supplement: Attachment — Submitted filename: Yu2025_response_to_reviewers.docx [file pbio.3003539.s016.docx]

We would like to thank both reviewers for their positive and constructive comments. In response, we have substantially revised our manuscript and believe it is substantially improved.

In response to Reviewer 2’s concerns that our findings may in part reflect inadequate capture of sequence variation in accessory genes by our feature set, we have included a new analysis of our pathological scenarios for *K. pneumoniae* using unitigs as predictive features. The results of the unitig analysis are consistent with our previous SNP-based analysis, indicating that our primary results are unlikely to reflect a problem with feature engineering.

We have also substantially revised our description of our results and the discussion in response to both reviewers and believe both the clarity and connection to previous work in comparative biology has been improved.

Reviewer #1: This manuscript tests the inaccuracy caused by phylogenetic dependency in machine learning-based detection of antibiotic resistance determinants. It is well known that taxa are not independent because they have traits that descend from common ancestors (this is also true for transferred traits, just the ancestry is different). The authors create cases where data is dependent in terms of phylogeny and show that predictors of antibiotic resistance are severely affected by this (even when positives are removed from clades). The phenomenon in itself is well known. But it seems the ML community is not very aware of it and this is a relevant cautionary tale.

We agree that the non-independence of related organisms is well-established. This manuscript grew out of our frustration in reviewing ML approaches to AMR prediction that did not consider phylogeny as a confounding factor, and not having a clear reference to point to that showed this also affects ML inference. We hope that our manuscript will encourage attempts to incorporate phylogenetic structure in ML applications to bacteria.

Some major comments.

- The literature on the topic of phylogenetic inertia is actually very old (much older than the GWAS methods cited in the text) and it would be relevant to connect to it. It dates from at least Felsenstein, American Naturalist, 1985. A recent review is Dewar, Nature Rev Genetics, 2025.

We thank the reviewer for highlighting the foundational literature on incorporating phylogenetic structure in inference. We now cite these references and others on lines 47-49, and again in the discussion lines 195-199.

- While the authors mention a few methods used in other contexts to control for phylogenetic structure, I found that part of the text to be superficial since it does not provide clear clues on how that could be used in more complex ML setups. Also, it seems to me important, in the spirit of the manuscript, to provide information on how one could make diagnostics that there is a problem on phylogenetic structure. This connects to the previous point and the existence of methods to assess phylogenetic dependence.

We have expanded our discussion of methods to control for phylogenetic structure on lines 195-199, including references to a number of articles discussing statistical methods for comparative analysis. Given the diversity of ML approaches from simple regression to deep learning methods, it is difficult to give general recommendations for how to incorporate phylogenetic structure. However, we have highlighted that phylogeny-aware cross-validation is one straight-forward tool that can be used to assess the generalizability of ML predictors.

Some parts of the text need to be easier to read for the average reader of PLoS Biology.

- For example, the presentation of the dataset used is too brief in the main text.

We have expanded the description of the dataset on lines 59-72 to provide additional details.

- The meaning of SHAP values is not explained.

We have added a brief description of SHAP values on lines 120-122.

- "Based on the clade structure of our data" is too succinct, since one has to go deeper in the data to understand how clades are defined (and even then it's implicit that clades are actually sequence types).

We have clarified on lines 73-77 that clades were defined based on deep branches in the phylogenetic tree constructed using core genome alignments and IQ-Tree, and that these clade definitions correspond closely to sequence types providing a biologically meaningful grouping consistent with established typing schemes.

- The results obtained about quinolonoes (difference between E. coli and S. aureus) are well known and published.

While there is an extensive literature on differences in quinolone resistance mutations in terms of both their fitness effects and the primary target of the quinolone (e.g. ParC vs. GyrA), we could not find a clear reference that would explain why we generally observe *parC* mutations but only *gyrA* in a single clade of *S. aureus*. We have now cited a review that discusses some of the variation observed in ciprofloxacin resistant mutants on lines 134-135 and added some speculation that this may reflect clade-specific differences.

Reviewer #2: In this paper, the authors aimed to identify and quantify the contributions of various well-documented and common biases present in pathogen sample collections used for training machine learning classifiers for AMR prediction. The underlying datasets included five bacterial pathogens (both Gram-negative and Gram-positive) with matched AMR-associated metadata. The primary analysis was structured to exclude sensitive/resistant isolates from subpopulations/clades of the bacteria to identify the model's ability to identify generalisable predictors. This was further supported by a meta-model analysis of the resulting models to identify the most important predictive model features, alongside a series of clade-based analyses and further clade-based models to identify the top AMR predictors and the proportion of predictors that overlapped between analyses (i.e., were generalisable signals). Overall, the authors have produced a well-rounded and well-argued manuscript that has quantified and clarified a number of potential confounders previously observed and discussed by a range of authors. It also provides a useful framework for future researchers to appropriately evaluate work in this area.

The manuscript is well-structured, and the analyses are both interesting and timely, considering the growing interest in this research area. The authors have provided scripts and supporting information that would allow for the re-analysis of the data, with some exceptions (see below). This paper would generally be of interest to researchers in the fields of antimicrobial resistance prediction, machine learning, and machine learning applied to bacterial genomic datasets, where these kinds of confounding biases are both common and problematic. It might also be of interest to public health researchers who are focused on the development of these models for the monitoring and prediction of AMR resistance from public health genomics datasets.

Overall, I feel that the authors' claims are mostly well supported by the evidence they have provided, but they have not accounted for some of the underlying methodological biases that may have influenced their findings. This is a concern, considering that the paper purports to address and account for biases in sample collection but have not considered that they may have introduced methodological biases. I would like to see some further analysis and/or discussion of the limitations of their approach or the impact that it might have had on their results and subsequent conclusions prior to publication.

We would like to thank the reviewer for their positive and constructive comments. We have performed additional analyses that attempt to assuage some of the reviewer’s concerns, and have also included additional discussion of the limitations of our approach.

Major Points:

1/ The authors have opted to take a hybrid approach to generating genomic variants for downstream analysis by machine learning. This entailed the generation of core genomic variants (mapping and variant calling) and accessory genome content presence-absence information (pangenomic analysis). Whilst this approach does aid interpretation, it has some methodological downsides in that it introduces biases:

1a/ Gene presence-absence information relies on a pre-processing tool (Roary) providing the 'correct' clustering and classification of accessory genes. However, accessory genome content may be highly variable both in its genomic proportion per species (presence/absence) and in the underlying genetic variation present within accessory genes. Some of the species discussed within the paper have significantly larger and more diverse accessory genomes than others (e.g., E. coli, K. pneumoniae). As genetic variation 'within' accessory genes might be an important feature, it would not be identified using this analysis. This should be discussed in the context of the literature and the analysis.

We agree that relying on Roary to cluster accessory genes into presence–absence patterns may not adequately capture genetic variation within accessory genes, and that the proportion and diversity of the accessory genome can vary greatly between species.

In response to this, we now include a limited analysis based on unitigs for 3 clades of *K. pneumoniae* in figure S10. The results are consistent with our SNP and presence-absence analysis, indicating that our observations are likely not a simple artifact of the sequence features we used. We have also added a brief discussion on lines 184-194 to acknowledge the importance of feature engineering in method development.

1b/ The mapping/variant calling analysis of the core genome will be biased by the amount of sequence considered 'core' within and between species. This may be particularly egregious in Klebsiella and E. coli, which have particularly large accessory genomes relative to their core genomes. The results of SF2-6 suggest that between-clade distance is an important factor in the resulting meta-model/clade-based analysis. The authors have not sufficiently addressed whether this is due to real clade-specific differences or the proportion of shared core genomic variation between clades. As the authors have provided no information on the reference genomes used for mapping or how the core genome was generated (i.e., whether it was performed once for the whole species or separately between clades), it is difficult to identify if this would be a limitation of their approach. The first scenario would entail a strict core, where important predictive features may be absent from their dataset; the second would involve more or less input information for some clade comparisons.

As the reviewer states, in our modeling, we included both SNPs called from the core genome alignments and accessory gene presence–absence data, thereby accounting for variation in both the core and accessory genomes. We have clarified in the methods (line 258) that the core genome was generated once per species using Roary, based on the complete dataset for that species, resulting in a strict core defined by genes present in all genomes. The analyzed genomes were either obtained from previously published studies or assembled de novo, as described in the “Data collection” section of the Methods. A reference genome was not used for mapping, we provide further details in response to Question 3 below.

Given our approach, we agree that the influence of between‑clade genetic distance observed in our meta‑model analysis may in part be due to differences in the proportion of shared core genomic variation between clades. We now briefly discuss this possibility on lines 176-181. However, we believe our limited unitig analysis shows that the definition of core v. accessory genomes is unlikely to be the primary factor driving our results in Figure 1.

2/ Further to the previous two points the authors might have instead considered instead taking an approach which is agnostic to bioinformatics pre-processing of their data, such as using k-mers or unitigs. I would like to see some discussion of the limitations of their methodological approach to genomic data in their discussion and perhaps some additional analyses of these potential biases on their outputs.

As mentioned previously, we now include a limited unitig analysis on lines 189-191. While the rest of our current analysis focuses on SNPs from the core genome and accessory gene presence–absence patterns, we have revised the Discussion to acknowledge this limitation and to specifically discuss unitig and k-mer approaches as alternatives.

3/ The authors have not documented which reference genomes they used for the SNIPPY-based variant calling. Without this documentation, it would be impossible to reproduce their analyses.

In our analysis, SNP-sites was applied to the core genome alignment generated by Roary, rather than mapping reads to an external reference genome. Therefore, no specific reference genome was required for SNP calling. We have clarified this in the main text on lines 67-69.

Minor:

1/ "Indicating that the ML models were conflating indicators of lineage with genuine indicators of AMR." — I am not sure this is fully supported by the analysis at this stage of the paper. The assumption here is that the drop in precision/recall is due to the model predominantly learning features that are indicators of lineage rather than indicators of resistance. However, we would assume that AMR elements are not necessarily distributed evenly across clades and that certain clades might have more, fewer, or uneven distributions of particular AMR genes or variants. In light of this, the drop in the percentage of resistance might instead be considered as the model having less relevant/important information to learn from. Later figures and analyses cover and discuss this further (i.e., Supp F9). I would suggest moving such a strong conclusion to later in the paper, where it is better supported by the evidence.

We agree that the conclusion that the models were conflating lineage‑associated markers with genuine AMR determinants is better supported later in the manuscript. In the revised manuscript, we have softened the interpretation in the Results section describing Fig. S7A to indicate this as a possible explanation, and we now present the stronger conclusion only after introducing the clade‑specific feature interpretation in Fig. S9, where the evidence directly supports it.

2/ There are some general formatting issues with some of the existing figures, which reduce their interpretability and effectiveness. In particular, Figure 1 is difficult to read. The colours selected do not have sufficient contrast and do not display well. Additionally, the figure legend is not particularly clear in context.

We thank the reviewer for this helpful feedback. In the revised manuscript, we have updated the colour scheme of Figure 1 to improve contrast, and we have refined the legend to provide clearer context for interpreting the figure.

3/ Figure 2: I am not sure if it is my screen but I literally couldn't see the bars in panels B+C. AUC 'socre' is incorrectly spelled.

We are also unsure if this is a problem with our figure file. In the revised manuscript, we have increased the size of the bars in panels B and C of Figure 2 to ensure they are clearly visible across different screens and corrected the spelling of “score.”

4/ Figure S2-6 - Again, the choice of colours did not visualise well in panel B.

We have revised the color scheme in panel B of Figures S2–S6 in line with our modifications to Figure 1.
